# The Invasive Mechanisms of the Noxious Alien Plant Species *Bidens pilosa*

**DOI:** 10.3390/plants13030356

**Published:** 2024-01-25

**Authors:** Hisashi Kato-Noguchi, Denny Kurniadie

**Affiliations:** 1 Department of Applied Biological Science, Faculty of Agriculture, Kagawa University, Miki, Kagawa 761-0795, Japan; 2 Department of Agronomy, Faculty of Agriculture, Universitas Padjadjaran, Jalan Raya Bandung Sumedang Km 21, Jatinangor, Sumedang 45363, Jawa Barat, Indonesia

**Keywords:** adaptation, allelopathy, asynchronous germination, defense function, flowering phenology, heteromorphic seed, natural enemy, phytochemical, plasticity

## Abstract

*Bidens pilosa* L. is native to tropical America and has widely naturized from tropical to warm temperate regions in Europe, Africa, Asia, Australia, and North and South America. The species has infested a wide range of habitats such as grasslands, forests, wetlands, streamlines, coastal areas, pasture, plantations, agricultural fields, roadsides, and railway sides and has become a noxious invasive weed species. *B. pilosa* forms thick monospecific stands, quickly expands, and threatens the indigenous plant species and crop production. It is also involved in pathogen transmission as a vector. The species was reported to have (1) a high growth ability, producing several generations in a year; (2) a high achene production rate; (3) different biotypes of cypselae, differently germinating given the time and condition; (4) a high adaptative ability to various environmental conditions; (5) an ability to alter the microbial community, including mutualism with arbuscular mycorrhizal fungi; and (6) defense functions against natural enemies and allelopathy. The species produces several potential allelochemicals such as palmitic acid, *p*-coumaric acid, caffeic acid, ferulic acid, *p*-hydroxybenzoic acid, vanillic acid, salycilic acid, quercetin, α-pinene, and limonene and compounds involved in the defense functions such as 1-phenylhepta-1,3,5-trine, 5-phenyl-2-(1-propynyl)-thiophene, 5-actoxy-2-phenylethinyl-thiophene, and icthyothereol acetate. These characteristics of *B. pilosa* may contribute to the naturalization and invasiveness of the species in the introduced ranges. This is the first review article focusing on the invasive mechanisms of the species.

## 1. Introduction

**Figure 1:**
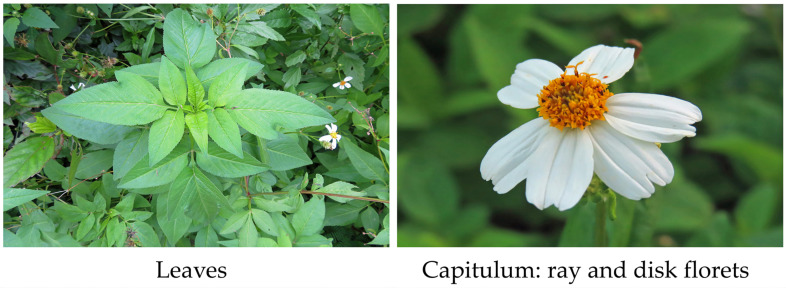
Leaves and capitulum of *B. pilosa*. The pictures were taken by the authors.

*Bidens pilosa* L., belonging to the Asteraceae family, is an annual (or biennial) herbaceous plant. The species grows 20–180 cm tall, and the stems are quadrangular with hairy straggling branches. It has alternate leaves with 3–5 pinnate leaflets, which are supported by a petiole (10–70 mm long). The leaflets are broadly ovate, serrate, and 30–70 mm long and 12–18 mm wide. Capitula occur at the end of the main stems and lateral branches and expand 5–12 mm in diameter. Capitula consist of 0–8 ray florets and 35–55 disk florets. The corollas of the ray florets are 7–15 mm long and white–yellow. The ray florets have poorly developed pistils and lack stamens. The disk florets have 3–5 mm long yellow corollas, five stamens, and well-developed 2–3 mm long pistils. Its fruits are black liner cypselae with 2–5 stiff awns of 2–4 mm long [1,2,3,4,5]. The species often forms thick monospecific stands [1,2,3] (Figure 1).

*B. pilosa* has been used as folk medicine in the treatment of various diseases such as fever, diarrhea, hepatitis, snake bite, and wounds [6,7] and as a nutritious vegetable [8,9,10,11]. Recent investigations showed *B. pilosa* has a wide range of pharmacological activity such as for malaria, cancer, diabetes, inflammation, and hypertension [12,13,14]. The leaves also contain high levels of protein [11,15].

Despite the pharmacological potential, *B. pilosa* is also known as a noxious invasive weed species. The species is native to tropical America and has naturized in over 60 countries of tropical, subtropical, and warm temperate regions in Europe, Africa, Asia, Australia, and North and South America [1,2,3]. Many of the invasive plant species potentially threaten the native flora and fauna in the introduced ranges [16,17,18]. *B. pilosa* has infested a wide range of habitats such as grasslands, forest margins, secondary forests, wetlands, streamlines, coastal areas, roadsides, railway sides, disturbed lands, pasture, plantations, and agriculture fields [1,2,19,20,21]. The species has also infested several islands such as Hawaii, Fiji, and the Cook Islands [22]. The ecosystems of these islands are very vulnerable to alien species [23,24]. *B. pilosa* is listed as a noxious agricultural and environmental weed in more than 40 countries [1,2,19,20,21]. According to the accumulated publications, the species is highly invasive and potentially causes serious negative impacts on natural ecosystems and agricultural crop production. However, there is no review article of the impacts and invasive mechanisms of *B. pilosa.* This paper provides an overview of the literature describing the impacts and invasive characteristics and mechanisms of the species. The species *B. pilosa* is divided into some varieties such as *B. Pilosa* var. *minor*, var. *pilosa*, and var. *radiata* [25,26]. Although some authors distinguished the varieties of *B. pilosa* for their investigations, many researchers did not distinguish them. Therefore, this paper describes the species as *B. pilosa* because it is impossible to confirm the variety of the species for their investigations. The literature was searched using a combination of the predominant online search engines, Scopus, ScienceDirect, and Google Scholar, and all possible combinations of *B. pilosa* with the following words: biology, invasive mechanism, habitat, impact, reproduction, flower, seed, plasticity, adaptation, nutrient, colonization, arbuscular mycorrhizal fugus, rhizobium, allelopathy, allelochemical, allelopathic substance, defense function, natural enemy, insecticidal activity, fungicidal activity, pharmacology, second metabolite, and global warming.

## 2. Impacts of *B. pilosa*

**Figure 2:**
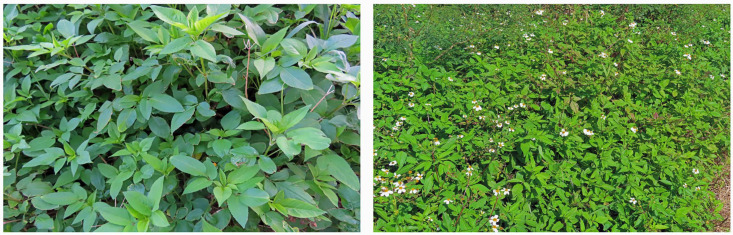
Monospecific stands of *B. pilosa.* The pictures were taken by the authors.

*B. pilosa* has the potential to rapidly grow and to form dense thickets. The species outcompetes crops in agricultural fields and eliminates indigenous plant species in introduced ranges by expanding the margins of its dense thickets [1,2,3,19,20,27]. *B. pilosa* was reported to replace the indigenous plant species of islands such as *Panicum fauriei* Hitcc. and *Scaevola coriacea* Nutt. on Hawaii [28], and *Salvia pleberia* R.Br. on Iriomote Island, Japan [29] (Figure 2).

*B. pilosa* infestation suppressed the growth of sugarcane (*Saccharum officinarum* L.) by 40% on day 60 and 80% on day 120 after sugarcane planting under field conditions in Okinawa, Japan, and caused 80% of the final production losses [30]. The species infestation reduced the production of a bean (*Phaseolus vulgaris* L.) by 48% in Uganda and by 18–48% in Peru [1]. The growth of this bean showed a significant negative correlation with the density of *B. pilosa* [31,32]. The species, at a density of 1.85 plants per m^2^, caused the bean yield to reduce by 18%, while 10 plants per m^2^ caused a reduction by 48% [33]. When *B. pilosa* and soybean (*Glycine max* (L.) Merr.) were grown together under field conditions, *B. pilosa* showed a higher relative growth rate than soybean and suppressed soybean’s vegetative growth and seed production [34]. A density of one and four plants of *B. pilosa* per m^2^, respectively, caused a soybean yield loss of 9.4% and 28% [2]. The contamination of the cypselae of *B. pilosa* also spoiled the quality of the crop grains [2]. When *B. pilosa* was germinated and grown with 16 crop plant species under different fertilizer and disturbance gradients under field conditions, *B. pilosa* showed a high competitive ability against crop plants from the families of Poaceae, Begoniaceae, Solanaceae, Balsaminaceae, Caryophyllaceae, and Convolvulaceae [27]. The stiff awns of its cypselae also irritate people and livestock [1,2]. Observations suggest that the infestation of *B. pilosa* causes a reduction in crop production and quality.

*B. pilosa* is also involved in the transmission of pathogens to agricultural crop plants as a vector. Sonchus yellow net virus was transmitted from *B. pilosa* to *Nicotiana glutinosa* L., *Nicotiana clevelandii* A.Gray, *Zinnia elegans* Jacq., and lettuce (*Lactuca sativa* L.) through the medium of the aphid (*Hyperomyzus lactuae* L.) [35,36]. *B. pilosa* has the potential to serve as a vector for tomato spotted wilt virus, which causes the typical tospovirus symptoms of chlorotic ringspots and necrosis on both young and older leaves of tomato (*Lycopersicon esculentum* Mill.) [37]. However, the movement of the virus between *B. pilosa* and tomato remains unknown. The papaya mealybug *Paracoccus marginatus* Williams & Granara de Willink, which is native to tropical America, was detected in South Asia, and *B. pilosa* was a host plant of the mealybug and transmitted it to other plant species [38]. The involvement of *B. pilosa* in the transmission of pathogens and insects may reduce the agricultural crop production.

As described in this section, the impacts of *B. pilosa* infestation on agricultural crop production were documented in several publications. However, its impacts on native plants and indigenous ecosystem are limited.

## 3. Fast Growth

*B. pilosa* established dense stands 50–135 days after its germination. The species starts flowering four–eight weeks after germination and produces mature seeds two–four weeks after flowering [39,40]. Regarding the population of the species in its favorite conditions, *B. pilosa* flowers throughout the year and makes up to four generations annually [1,2,5]. *B. pilosa* showed a higher growth rate, leaf area, and biomass than two indigenous congeners, *Bidens bripartit* (Lour.) Merr. & Sherff and *Bidens tripartita* L., under field conditions in China [41]. *B. pilosa* also showed more efficient utilization of water, photosynthetic fixed carbon, and nitrogen than the indigenous *Cirsium setosum* (Willd.) M. Bieb [42]. *B. pilosa* was reported to regenerate from remaining stems after cutting the above-ground parts of the plants [29]. These observations suggest that *B. pilosa* rapidly grows, establishes dense stands soon after germination, and produces several generations in a year.

## 4. Reproduction

**Figure 3:**
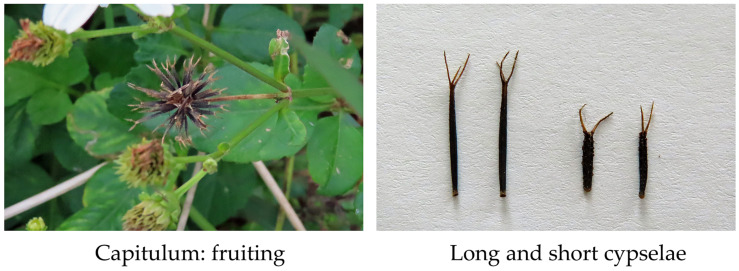
Capitulum and cypselae of *B. pilosa.* The pictures were taken by the authors.

The species produces about 80 capitula on long peduncles arising from the apexes of the stems and the leaf axils. A single capitulum contains ray and disk florets, as explained in the Introduction. The ray florets are sterile but serve as a nectar guide for pollinators. A nectary gland is present at the base of the style of disk florets [5]. The fertilized disk florets produce single-seeded black cypselae within four weeks [1,2,3,4]. Each plant produces 2000–6000 cypselae during its life cycles [1,2,43]. The species produces two types of cypselae within the same capitulum: long cypselae (8–10 mm in length) and short cypselae (3–7 mm). The production rate of the long and short cypselae was 64% and 36%, respectively [5,44]. Relatively young plants produced more long cypselae, and senescent plants produced more short cypselae [44,45]. Both types of cypselae have 2–5 stiff awns 2–4 mm long and can be easily dispersed by the attachment of the awns to animals, birds, and human clothes or by wind and water [5,43]. The seeds in cypselae remain viable for 5–6 years [2] (Figure 3).

Long cypselae have thin seed coats compared with short cypselae. Long cypselae germinated soon after their dispersion under a wide range of conditions, while short cypselae showed dormancy and germinated only under favorable conditions, such as adequate moisture and temperature, for the subsequent growth of seedlings [5,45,46]. The germination of short cypselae was stimulated by red light and gibberellin application [46], which indicates phytochrome involvement in the germination. Long cypselae showed no light sensitivity and germinated in both light and dark conditions [45]. Long cypselae produced three–four generations in a year due to their quick germination after dispersion, while short cypselae germinated in subsequent years due to their dormancy [5]. Long cypselae contribute to the species by continuously increasing the population, while short cypselae contribute to the species by remaining in reserve for the population [5,47].

The *B. pilosa* population also shows two phenological types of flowering–fruiting events during the growing season. Less than 10% of the population of the species flower one–two months after germination (early type), and the others flower four months after germination (normal type). Normal-type plants grow larger than early-type plants, and early-type plants produce fewer and heavier seeds than normal-type plants. The seeds from early-type plants more quickly germinate than those of normal-type plants, and both seeds do not show dormancy. The different flowering phenology affects seed mass and production and germination. However, the seeds from both types of plants produce early-type and normal-type offspring. Therefore, the flowering–fruiting events of their offspring are not affected by the parental types [48]. Early-type plants may contribute to the species quickly expanding because of their quick growth and flowering–fruiting. Normal-type plants may contribute to the species by stably increasing the population and/or maintaining the population.

The germination behavior of plants affects plant fitness and persistence. Invasive plant species often show earlier and/or rapid germination and asynchronous germination. The production of different biotypes of seeds by a single plant is also one of the characteristics of invasive plant species [49,50,51,52]. Early-germinating species and rapidly germinating species may benefit from less competition with other plant species for resources and niches [53,54]. These species enable the suppression of the germination and establishment of later-germinating species [51,52,55]. However, early and rapid germination is a risky strategy under unpredictable environmental conditions, especially in the early growing season, and often increases seedling mortality in such conditions [51,56,57]. Asynchronous germination may be more beneficial to the rapid expansion of species under relatively stable conditions and to the persistence and survival of species under unpredictable environmental conditions, regarded as a significant characteristic of invasive species to expand their ecological niches and population [50,58].

*B. pilosa* produced a great number of cypselae during its life cycle, different biotypes of cypselae such as long cypselae and short cypselae [5,43,45,46], and different phenological types of flowering–fruiting events such as the early-type flower and normal-type flower [48]. The asynchronous germination of *B. pilosa* is a strategy in which one of the seeds’ subsets may successfully germinate and establish itself at different times and under different conditions, providing the chance to survive and colonize in new habitats. Therefore, *B. pilosa* may be able to survive as a widespread invasive species in different habitats and to expand its distribution in introduced ranges.

In addition, *B. pilosa* showed different breeding systems at a variety of levels within the species. *B. pilosa* var. *minor* and *B. pilosa* var. *pilosa* produced 89% and 74% of seeds, respectively, compared to the corresponding open-pollinated capitula, although 73% of the seeds were set in open-pollinated capitula [25,37]. This observation suggested that *B. pilosa* var. *radiata* is highly self-incompatible, whereas the other two are self-compatible. Therefore, *B. pliosa* showed different breeding systems at a variety of levels within the species. The invasiveness of *B. pilosa* var. *radiata* was observed to be higher than that of *B. pilosa* var. *minor* and var. *pilosa.* When solar irradiation is high, *B. pilosa* var. *radiata* allocates more biomass to axillary shoots than the other two varieties, contributing to the expansion of the varieties [26]. Xenogamy enables the species to increase its genetic diversity, which favors its establishment in heterogenous and variable environments [5].

## 5. Adaptation

*B. pilosa* grows best in areas with full sunlight, a mean annual temperature between 25 °C and 38 °C, and annual rainfall between 500 mm and 3500 mm. It can grow in a wide range of soil types including sand and lime soil with a pH ranging from 4 to 9 and salinity up to 100 mM NaCl. The species tolerates frost, and the regeneration of its roots occurs after temperatures as low as −15 °C [1,2]. The species benefits from disturbances such as fire and soil tillage and quickly infests after a disturbance [19,59].

Under resource-limiting conditions, some invasive plant species can outcompete indigenous plant species either by efficient resource uptaking or by a lower resource requirement, called the resource conservative strategy [60]. The carbon (C), nitrogen (N), and phosphorus (P) contents in plant tissues and their ratios such as N:P and C:P reflect the use and adjustment of the available nutrients and the relative growth rate of the plants [61,62,63]. Lower N:P and C:P ratios in plant tissues indicate a higher P content in the whole plants and the stimulation of protein synthesis, resulting in higher growth and reproductive output [61]. A higher C:N ratio indicates higher C assimilation and lower nutrient requirements [63]. *B. pilosa* under low-nutrient conditions showed a higher C:N ratio in the roots, indicating a lower nutrient requirement and higher C assimilation, adapting a resource conservative strategy. *B. pilosa* under high-nutrient conditions showed lower N:P and C:P ratios in the roots, indicating higher protein synthesis, growth, and reproductive output [64]. When maize (*Zea mays*, L.) and *B. pilosa* grew together under field conditions, the C:N, C:P, C:K, N:P, and N:P ratios in *B. pilosa* significantly increased, while those ratios in maize changed a little, which indicates that *B. pilosa* has a distinct survival strategy under nutrient-competitive conditions, and is able to more efficiently use nutrients [65]. Therefore, *B. pilosa* may apply a competitive strategy in nutrient-rich environments and a resource conservative strategy in resource-poor environments.

*B. pilosa* showed a similar plant height as the indigenous plant species *B. biternata* (Lour.) Merr. & Sherff and *B. tripartita* L. under unfavorable light and moisture conditions. However, *B. pilosa* showed a greater plant height, biomass, and growth rate than both indigenous species under favorable light and moisture conditions. *B. pilosa* allocated more resources to the root biomass under a high-light condition and to the leaf biomass under a low-light condition [41]. When *B. pilosa* and *Bidens biternata* were grown in the conditions of different light intensities (40% and 10% sunlight) for 64 days, *B. pilosa* showed a higher leaf mass, a higher total leaf area, and increased photosynthesis than *B. biternata* [66]. These observations suggest that *B. pilosa* has a higher adaptative ability and a higher phenotypic plasticity than *B. biternata* and *B. tripartita* under low-light and low-moisture conditions, which may contribute to the invasiveness of *B. pilosa*.

Extreme precipitation often causes short-term waterlogging conditions for terrestrial plants, which cause hypoxia and serious damage to the plants’ root systems [67,68]. Short-term waterlogging stimulated adventitious root generation and higher dehydrogenase activity in the roots of *B. pilosa* [69,70], which is the adaptive response to the hypoxic condition [67,68]. The reduction in the photosynthesis and growth rate of *B. pilosa* under waterlogging conditions was significantly less than that of an indigenous species, *B. biternata* [70]. Therefore, *B. pilosa* may tolerate waterlogging conditions better than the indigenous species *B. biternata.*

The seeds of *B. pilosa* were collected from three different locations in China. Those seeds germinated at a temperature between 10 °C and 30 °C. The seeds lost viability after 8 days of continuous heating at 40 °C or 30 min heating at 50 °C. However, the intraspecific variation for high-temperature tolerance was found among seeds from different collection sites [71]. The germination requirements for temperature and water also significantly differed among seeds from different collection sites [72]. Seeds obtained from nine locations in Brazil showed 20–97% germination rates at a temperature of 10 °C–35 °C. The seed weight and the rates of germination and dormant seeds significantly varied among collection locations [73]. The germination rate of *B. pilosa* was higher with seeds buried in shallow soil than deep soil, and the seed dormancy was greater with deeper soil than shallow soil [74,75]. Therefore, the population of *B. pilosa* from different locations showed different tolerance and requirements for germination such as temperature and water and the different ratios of dormant seeds. The variation in the water requirement, temperature tolerance, and dormancy among seeds from different locations may be involved in the adaptation of *B. pilosa* to local conditions.

These observations suggest that the acclimation ability of *B. pilosa* to various environmental conditions such as soil fertility, temperature, and solar radiation is high. It also adapts to waterlogging conditions.

## 6. Genetic Variation

The species was divided into three varieties such as *B. pilosa* var. *minor*, var. *pilosa*, and var. *radiata*, and these varieties showed different morphological traits and adaptive potentials [25,26]. The genetic variation of five loci such as the chloroplast and nuclear ribosomal DNA of *B. pilosa* var. *minor*, var. *pilosa*, and var. *radiata* collected in Taiwan was found to be high, and each of these varieties showed different morphological traits and adaptive potentials [76]. However, those genetic variations have not been related to the invasiveness of the species. The genetic variation of plastomes in the Chinese population was very high, to the degree that specimens from the same population were misidentified as different species [77]. The genetic variations of *B. pilosa complex* were also determined for the identification of the species [78]. However, information on the genetic variation of the species is very limited. In addition, there is no information available for the genetic variation and invasiveness of the species.

## 7. Effects on Microbial Community

Invasive plants often change the soil condition and alter the soil microbial community, affecting the carbon and nitrogen cyclings, soil organic matter, and decomposition rate of the plant residues [79,80,81,82,83,84,85,86]. The rhizosphere of *B. pilosa* develops different soil microbial assemblages compared with indigenous plant species [87]. *B. pilosa* and the indigenous plant species *Saussurea deltoidea* (D.C.) C.B.Clark were grown in non-sterile and sterile soil obtained from under dominant stands of a native shrub, *Dodonaea viscosa* (L.) Jacq. *B. pilosa* showed a greater biomass in non-sterile soil than *S. deltoidei*, and soil sterilization had a more negative effect on the growth of *B. pilosa* than *S. deltoidei* [88]. These observations suggest that the soil microbiota under *D. viscosa* affects *B. pilosa* more than *S. deltoidei*, and *B. pilosa* may benefit in introduced ranges by using the native soil microbiota associated with *D. viscosa.* The nitrogen and potassium contents in the soil under *B. pilosa* were significantly higher than those under *Bidens bipinnata* L. under the condition of full sunlight [89], indicating that *B. pilosa* is involved in rapid nutrient mobilization in the full-sunlight condition.

Arbuscular mycorrhizal fungi (AMF) promote their host plant’s fitness by increasing water and nutrient acquisition and the defense performance against pathogen attacks and stress conditions [90,91,92,93,94]. The inoculation of AMF, *Septoglomus vicosum*, *S. constrictum*, and *Glomus perpusillum*, to *B. pilosa* increased its competitive ability, and AMF-inoculated *B. pilosa* outcompeted an indigenous plant species, *Setaria viridis* (L.) P.Beauv. The plant community in areas has finally been replaced by *B. pilosa* [95]. The inoculation of AMF, *Funneliformis mosseas*, *Diversispora versiformis*, and *Glomus diaphnum* and/or *G. etunicatum*, increased the biomass and the absorption ability of nitrogen and phosphorus of *B. pilosa* in a karst environment [96,97]. The inoculation of *Glomus mosseae* also increased the drought stress tolerance of *B. pilosa* [98]. Therefore, the mutualism of *B. pilosa* with AMF may increase its competitive ability and nutrient acquisition, resulting in a greater biomass of *B. pilosa* and the elimination of native plant species.

These observations suggest that *B. pilosa* may alter the soil microbial community and/or utilize the native soil microbiota. These microbes and their mutualism with AMF may contribute to increase the ability of *B. pilosa* regarding competition, nutrient acquisition, and stress tolerance.

## 8. Allelopathy

The interaction of introduced plants with indigenous plant species is one of the important determinants of the success of their naturalization in introduced ranges [99,100,101,102]. Invasive plants were often reported to have an ability of allelopathy. Allelopathy is the chemical interaction between donor plants and receiver plants, and chemicals involved in allelopathy are defined as allelochemicals [100,103]. Allelochemicals are released from donor plants into neighboring environments through volatilization, rainfall leachates, root exudation, and the decomposition of donor plant residues in the rhizosphere soil. Allelochemicals disturb the germination, growth, and regeneration process of neighboring plant species, and many invasive plants showed allelopathic activity [104,105,106]. Allelopathic plants synthesize and store allelochemicals in certain plant tissues until releasing them into neighboring environments [107,108,109,110,111]. Therefore, several researchers investigated the allelopathic activity and allelochemicals in extracts from different plant tissues and the residues of *B. pilosa* and its rhizosphere soil.

The chopped leaves of *B. pilosa* were incorporated into a rice paddy field (2 tons per hectare) in Thailand. The treatments suppressed the total emergence number and weight of 12 weed species by 15% and 18%, respectively, compared with the control, 30 days after the treatments. The major weeds in the area were *Brachiaria mutica* (Fordk.) Stapf., *Commelina diffusa* Rurm., *Monochoria vaginalis* Presl, and *Marsilea quadrifolia* L. *B. pilosa* suppressed the emergence total weed number by 4, 0, 35, and 31% of the control, and the total weed weight by 17, 0, 88, and 3% of the control for *Brachiaria mutica*, *Commelina diffusa*, *Monochoria vaginalis*, and *Marsilea quadrifolia*, respectively [112]. The mulching and incorporation of whole plants of *B. pilosa* into soil suppressed the weed emergence and density under greenhouse conditions [113]. The residues of the shoots and roots of *B. pilosa* incorporated into the soil suppressed the growth of *Cyperus rotundus* L. Even the soil collected under the *B. pilosa*-infested field also suppressed the growth of *C. rotundus* [114]. These investigations suggested that certain allelochemicals may be released into the soil during the decomposition process of the leaves, shoots, and roots of *B. pilosa* and suppress the emergence and growth of these weed species.

The continuous application of the root exudate of *B. pilosa* by a recirculation system suppressed the seedling growth of lettuce, bean, maize, and *Sorghum bicolor* (L.) Moench. The dry mass of lettuce, bean, maize, and *Sorghum bicolor* was reduced by 38%, 55%, 47%, and 58% on day 14, respectively [115]. The root exudate of *B. pilosa* also inhibited the germination and growth of *Leucaena leucocephala* (Lam.) de Wit., *Echinochloa crus-galli*(L.) P. Beauv., *Medicago sativa* L., and rice (*Oryza sativa* L.) [116]. When *B. pilosa* and *Cyperus rotundus* were grown together under pot conditions, the growth of *Cyperus rotundus* was suppressed. However, when activated carbon was added into the cultivation soil in the pots, the suppression was significantly reduced [114]. These investigations suggested that certain allelochemicals may be released into the soil as the root exudates of *B. pilosa*, and suppress the germination and growth of these plant species.

The spore germination, growth, and photosynthesis of the fern species *Pteris multifida* Poir. were also suppressed by the root exudation of *B. pilosa.* The root exudation increased the antioxidant enzyme activities in the gametophytes of the ferns such as superoxide dismutase, catalase, glutathione reductase, and glutathione *S*-transferase, which resulted in the death of the gametophytes. Palmitic acid (hexadecanoic acid; fatty acid) is a major compound in root exudates [117,118]. However, it is not certain if palmitic acid is involved in the inhibitory effect of the root exudate of *B. pilosa* because the allelopathic activity of this compound has not been proved.

Aqueous extracts of the whole parts of *B. pilosa* inhibited the germination of *Amaranthus dubius* Mart. ex. Thell [119] and the root growth of *Ageratum conyzoides* L. [120]. Fresh-cut *B. pilosa* (1 cm in length) or its aqueous extract was applied with irrigation water to *Echinochloa crus-galli* seedlings grown in pots (20 cm d.i.; 5 kg of soil). Both treatments resulted in increasing the mortality of *E. crus-galli*. When fresh cuts of *B. pilosa* were also applied to the rice paddy field with irrigation water, the treatment suppressed the weed emergence of the common weeds in rice fields such as *E. crus-galli*, *E. coloa* (L.) Link., *Leptochloa chinensis* (L.) Nees, *Ludwigia hyssopifolia* (G.Don) Exell, *Sphenoclea zaylania* Gaerth, *Cypens iria* L., *Fimbristylis dichotoma* (L.) Vahl., and *Fimbristylis milacea* (L.) Vahl. The treatments significantly increased rice production due to this inhibition of weed emergence [113,121]. These observations suggested that aqueous extracts of *B. pilosa* may contain certain allelochemicals and that these allelochemicals suppress the germination and growth of these plant species. Some allelochemicals may also be released by rainfall leachates because of the allelopathic activity of aqueous extracts and the irrigation water with fresh cuts of *B. pilosa*.

Boiling water extracts of the leaves, stems, and roots of *B. pilosa* inhibited the germination and growth of *Raphanus sativus* L. and *Echinocholoa crus-galli.* Phenol derivatives such as *p*-coumaric acid, caffeic acid, ferulic acid, *p*-hydroxybenzoic acid, vanillic acid, and salycilic acid were identified in the extracts [122]. These compounds were found in several other plant species and showed germination and growth inhibitory activity as allelopathic agents [123,124,125]. The inhibitory activity of those compounds was considered to be due to the disturbance of nutrient uptake, water transport, and photosynthesis [126,127,128]. Salycilic acid is also involved in the plant defense function against pathogen attacks [129].

Quercetin (flavonoid) was identified in the leaf extracts of *B. pilosa* [130]. Quercetin was reported to inhibit the growth and mitochondrial function of several plant species as an allelopathic agent [131,132,133,134]. Several terpenes were identified in the essential oil of *B. pilosa* [135]. Terpenoids were reported to be involved in the defense function of plants such as anti-fungal, anti-bacterial, and anti-feeding activities, in the interaction with insects such as for pollination, and the attraction of predators of their natural enemies [136,137,138,139,140,141]. Among the identified terpenoids found in *B. pilosa*, α-pinene and limonene, which are readily volatile, showed allelopathic activity and inhibited the germination and growth of several plant species [123,142].

**Figure 4:**
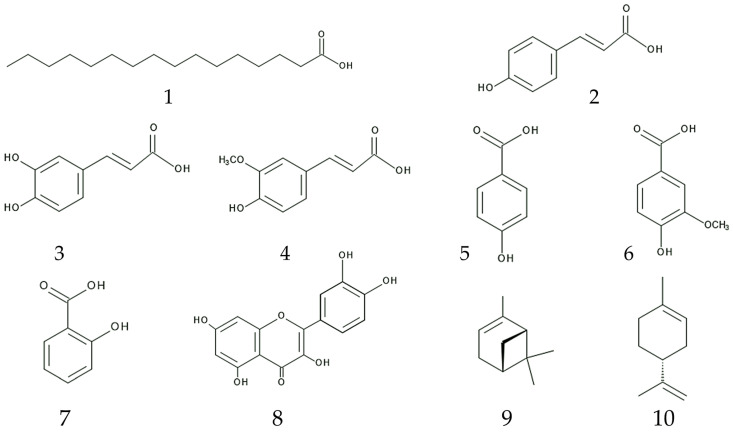
Possible allelochemicals in *B. pilosa.* **1**: palmitic acid; **2**: *p*-coumaric acid; **3**: caffeic acid; **4**: ferulic acid; **5**: *p*-hydroxybenzoic acid; **6**: vanillic acid; **7**: salycilic acid; **8**: quercetin; **9**: α-pinene; **10**: limonene.

These investigations suggested that whole parts of *B. pilosa* may contain water-extractable allelochemicals, and some of them may be released into its rhizosphere soil through the volatilization, rainfall leachate, root exudation, and decomposition processes. The novel weapons hypothesis indicates that the competitive ability of invasive plants is high due to allelochemicals (weapons) [99,100,103]. Therefore, these allelochemicals may be effective on indigenous plant species to suppress their regeneration process through germination and growth inhibition and contribute to the invasion of *B. pilosa* in introduced ranges (Figure 4).

## 9. Nematocidal, Fungicidal, and Insecticidal Activity

The interaction between introduced plants and natural enemies such as herbivore insects and pathogens is also one of the important determinants of the success of naturalization in introduced ranges [143,144,145]. When aqueous extracts of the leaves and roots of *B. pilosa* were applied into soil that contained the earthworm *Eisenia fetida* Savigny, the treatments resulted in the suppression of the body mass and respiration of the earthworm and in the enhancement of the oxidative- and DNA-damage biomarkers in the earthworm, in a concentration-dependent manner [146]. The aqueous extracts of the above-ground parts of *B. pilosa* increased the mortality of the root-knot nematode *Meloidogyne incognita* Kofoid & White, the pine-wood nematode (*Bursaphelenchus xylophilus* (Steiner & Buhrer) Nickle), and the pine sawyer (*Monochamus alternatus* Hope), which is the vector of the pine-wood nematode, with the extract concentration-dependent manner [147,148]. The boiling water extracts of the leaves, stems, and roots of *B. pilosa* showed the growth-inhibitory activity on the pathogenic fungi *Corticium rolfsii* (Curzi) C.C. Tu & Kimbr., *Fusarium solani* (Mart.) Sacc., and *F. oxysporum* Schlecht. emend.Snyder & Hansen [122].

The methanol leaf extracts of *B. pilosa* showed insecticidal activity against three pests, the rice weevil (*Sitophilus oryzae* L.), grain beetle (*Oryzaephilus surinamensis* L.), and bean weevil (*Acanthoscelides obtectus* Say), with the extract concentration-dependent manner [149]. The spray of the aqueous extracts of *B. pilosa* reduced pest species such as flower beetles (*Epicauta albovittata* Gestro and *E. limbatipennis* Pic), foliage beetles (*Ootheca mutabilis* Sahlberg, and *O. bennigseni* Weise), and pod suckers (*Clavigralla tomentosicollis* Stäl and *C. schadabi* Dollong) [150]. Four polyacetylenes, 1-phenylhepta-1,3,5-trine, 5-phenyl-2-(1-propynyl)-thiophene, 5-actoxy-2-phenylethinyl-thiophene, and icthyothereol acetate isolated from the above-ground parts of *B. pilosa* showed insecticidal activity against the larvae of six moth species, *Plutella xylostella* L., *Spodoptera litura* Fabricius, *Mythimna separata* Walker, *Spodoptera exigua* Hübner, *Helicoverpa armigera* Hübner, and *Ostrinia furnacalis* Guenée, with LD_50_ values ranging between 0.133 and 4.084 μg per larva. However, 1-phenylhepta-1,3,5-trine, among the four polyacetylenes, showed the highest insecticidal activity [151]. 1-phenylhepta-1,3,5-trine (phenylheptatriyne) also showed nematocidal activity [152] and allelopathic activity against the germination and growth of *Asclepias syriaca* L., *Chenopodium album* L., *Phlem pratense* L., and *Trifolium pratense* L. [153].

**Figure 5:**
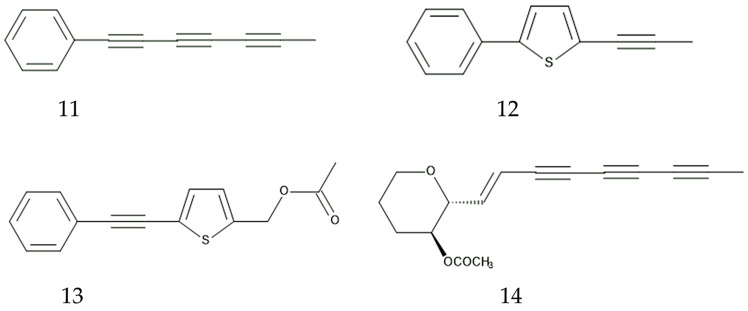
Possible compounds involved in the defense function of *B. pilosa.* **11**: 1-phenylhepta-1,3,5-trine, **12**: 5-phenyl-2-(1-propynyl)-thiophene, **13**: 5-actoxy-2-phenylethinyl-thiophene, **14**: icthyothereol acetate.

These investigations suggested that *B. pilosa* contains water and/or methanol extractable compounds that have nematocidal, fungicidal, and insecticidal activity. These compounds may contribute to the increase in the fitness of *B. pilosa* and be involved in its invasiveness in introduced ranges (Figure 5).

## 10. Secondary Metabolites

Recent pharmacological investigations showed that *B. pilosa* contains more than 200 natural compounds, in many chemical classes such as fatty acids, polyacetylenes, flavonoids, terpenoids, phenolics, and their glycosides. Some of these compounds showed pharmacological activity such as anti-malaria, anti-cancer, anti-diabetic, anti-allergic, anti-hypertensive, and anti-inflammatory activity [11,14,154,155,156,157,158]. Although most of the identified compounds in *B. pilosa* have not yet been related to its invasiveness, some of these identified compounds may be involved in allelopathy and defense functions against herbivores and pathogens. As described in Section 7 and Section 8, the extracts, root exudates, residues, and rhizosphere soil of *B. pilosa* showed allelopathic activity and nematocidal, fungicidal, and insecticidal activity. However, the entirety of the compounds involved in these activities may not be identified. Therefore, some of these compounds and/or unidentified compounds in *B. pilosa* may be involved in allelopathy and defense functions against herbivores and pathogens. In fact, several secondary metabolites in invasive plants showed multiple functions such as anti-herbivore, anti-fungal, and allelopathic activity [100,102,159,160,161]. Some of these compounds in *B. pilosa* may contribute to the invasiveness and naturalization of species in introduced ranges.

## 11. Prospective

The impact of biological invasions with global warming has been identified as a serious threat to biodiversity on the planet [162,163,164]. The increasing mean annual temperature promotes the invasion of alien species originating from warmer regions [165,166]. Warmer conditions enhance the competitive ability of alien plant species [167]. The growth and reproduction of *B. pilosa* was enhanced in warmer conditions by the shifting of its phenology to early germination and flowering and prolonged vegetative growth and reproduction [168]. Global warming trends may favor the spread of *B. pilosa* northward in the Northern Hemisphere and southward in the Southern Hemisphere, originating from warmer regions. The species will possibly expand into these areas in the near future and may cause serious threats to the natural ecosystems in the areas.

Several natural enemies of *B. pilosa* have been recorded in its native ranges such as *Cercospora bidentis* Tharp, *Ralstonia solanacearum* Smith, *Sclerotinia sclerotiorum* (Lib.) de Baary, *Bidens* mosaic virus, and Sonchus yellow net virus [2,169,170,171]. However, biological control agents for *B. pilosa* have not yet been sufficiently investigated [2]. The management of *B. pilosa* can be achieved with several herbicides such as glyphosate, atrazine, 2,4-D glyphosate, imazethapyr, metribuzin, and paraquat [2,75,172,173]. However, several herbicide-resistant biotypes of *B. pilosa* against glyphosate, atrazine, and imazethapyr were reported [174,175]. The appearance of multiple herbicide-resistant biotypes is a serious issue for weed management. The most effective management strategy for herbicide-resistant weeds is probably to rotate the herbicide’s sites of actions [176,177]. The employment of herbicide mixtures and the rotation of different types of herbicides in different growing seasons may be one of the options to control the herbicide-resistant biotypes of *B. pilosa* [178,179].

## 12. Conclusions

An annual or biennial plant species, *B. pilosa* quickly grows and has several generations during a growing season. Each plant produces 2000–6000 cypselae during its life cycles. There are different biotypes of cypselae: quick germination after dispersion, late germination with dormancy, early flowering–fruiting types, and normal flowering–fruiting types. Asynchronous germination and flowering–fruiting may be beneficial for the persistence and survival of the species under unpredictable environmental conditions and for rapidly increasing the population under relatively stable conditions. *B. pilosa* showed a highly adaptative ability regarding water, light, and nutrient availability, temperature, and flooding. The populations of *B. pilosa* from different locations showed different requirements for germination such as temperature and water and different ratios of dormant seeds. *B. pilosa* altered the microbial community in infested areas, and mutualism with arbuscular mycorrhizal fungi increased the competitive ability of *B. pilosa*. The species showed allelopathic activity and contains several allelochemicals, which suppress the germination and growth of other plant species. *B. pilosa* also showed defense functions against pathogen fungi, nematodes, and insects and contains compounds involved in the functions. These characteristics of *B. pilosa* may contribute to the naturalization and invasiveness of the species. Global warming trends may also favor the spread of this species into additional non-native areas and may increase the threat of the species.
